# Trajectory-Based Morphological Operators: A Model for Efficient Image Processing

**DOI:** 10.1155/2014/801587

**Published:** 2014-04-14

**Authors:** Antonio Jimeno-Morenilla, Francisco A. Pujol, Rafael Molina-Carmona, José L. Sánchez-Romero, Mar Pujol

**Affiliations:** ^1^Departamento de Tecnología Informática y Computación, Universidad de Alicante, P.O. Box 99, E-03080 Alicante, Spain; ^2^Departamento de Ciencia de la Computación e Inteligencia Artificial, Universidad de Alicante, P.O. Box 99, E-03080 Alicante, Spain

## Abstract

Mathematical morphology has been an area of intensive research over the last few years. Although many remarkable advances have been achieved throughout these years, there is still a great interest in accelerating morphological operations in order for them to be implemented in real-time systems. In this work, we present a new model for computing mathematical morphology operations, the so-called morphological trajectory model (MTM), in which a morphological filter will be divided into a sequence of basic operations. Then, a trajectory-based morphological operation (such as dilation, and erosion) is defined as the set of points resulting from the ordered application of the instant basic operations. The MTM approach allows working with different structuring elements, such as disks, and from the experiments, it can be extracted that our method is independent of the structuring element size and can be easily applied to industrial systems and high-resolution images.

## 1. Introduction

During the last 10–15 years, many different improvements have been proposed in order to implement morphological operations in a more efficient way than the original Serra's algorithm [1]. The methods for increasing the speed of morphological operators can be divided into two main groups: (i) algorithms that reduce the computation time of the morphological filters through the decomposition of the structuring element into smaller sets and (ii) algorithms that eliminate the inherent redundancies in the calculation of the morphological operations, achieving, as a consequence, an increase in the processing speed of such operations.

To a large extent, the most popular morphological methods to achieve this speed enhancement are within the first group. One of the most important ones is the van Herk and Gil-Werman's (HGW) algorithm [2, 3], which is among the fastest methods in order to implement erosions and dilations in gray-tone images, having a computational complexity independent of the size of the structuring element. In general terms, this method is designed for 1D elements and works with linear structuring elements composed of horizontal and/or vertical segments. Its main strength comes from the fact that only 3 comparisons are needed to obtain the output pixel. In [4] an improvement of this algorithm was made, achieving only 1.5 comparisons per output pixel but increasing significantly the computational complexity of the method, since it implies the generation of ordered lists. HGW's method has had a great impact since it was first proposed and, up to now, a great amount of works continue to develop new improvements of this algorithm, many of them include some kind of hardware optimization [5–7].

In [8], authors extended the HGW's model to the case of using lines with arbitrary orientations. In addition, in their work from 2001 [9], they improved their model so that two-dimensional structuring elements are also taken into account, making use of recursion. An interesting set of extensions were also presented in this paper, among which they include a method to approximate discrete disks by using cascades of dilations with periodic lines.

On the other hand, an algorithm for calculating grayscale morphological operations with flat, arbitrary-shaped structuring elements was presented in [10]. Their approach is independent of both the image content and the number of necessary gray levels. The use of arbitrary elements is interesting, particularly in cases where a structuring element cannot be decomposed into smaller ones. Essentially, the algorithm decomposes a structuring element into a series of chords that can be understood as a series of pixels of maximum extent and considers each chord as a horizontal structuring element. It also uses a look-up table to store the minimum intensity values of the pixels belonging to each chord. The experiments were performed with a wide variety of elements and showed that this method improves many others that were developed to decompose some specific types of structuring elements. It also allows working with floating point data.

Another improvement in the decomposition of arbitrary-shaped structuring elements has been proposed in [11]. The decomposition method is recursive and is optimized by using genetic algorithms, thus improving the results of other well-known decomposition approaches based on genetic algorithms, such as the ones described in [12–14].

In relation to this, a mathematical morphology algorithm for spatially variant square structuring elements was developed in [15], achieving very low temporal cost and memory requirements. In addition, they proposed an efficient hardware implementation of morphological operations based on this type of structuring elements; this hardware architecture is presented as a hardware accelerator for the dilation and erosion operations in embedded systems.

Recently, in [16] a method for the development of morphological filters that runs in linear time with respect to the image size and is constant in time with respect to the size of the structuring element was proposed. It is based on the decomposition of a rectangular structuring element into one-dimensional segments, then it eliminates redundant values and, finally, the result is encoded by calculating the distance between every change of the value. The authors claim that it is possible for this method to achieve an efficient real-time implementation, having also very small memory requirements and supporting floating-point data.

Regarding the algorithms to eliminate redundancies, the one proposed by [17] is particularly interesting, which introduced the concept of anchor, defined as the position in which a signal *f* is not affected by the application of a certain operator Ψ. Their approach is based on the search for anchors for the erosion and the closing, and it allows a morphological operation to run with one-dimensional structuring elements about 30% faster than previous methods. However, the main drawback is that this method requires the use of histograms, so that its extension to two and three dimensions is not straightforward and the improvement in computation speed is therefore minimized. In addition, the algorithm depends greatly on the image content. Another significant work is described in [18], which is focused on image binarization using morphological operators. To do this, the so-called quick-closing and quick-opening are defined; these operations have reduced computational cost and remove the redundant comparisons in the neighborhood of every pixel. The method works efficiently, but only for square-shaped structuring elements.

To sum up, from this revision two conclusions emerge: first of all, there is still a great interest from many research groups in order to accelerate morphological operations, both for improving the basic morphological algorithms and for their hardware implementation; on the other hand, most of these investigations are based on optimizing morphological filters for those cases where there is a dependence on the shape of the structuring element, with the square/rectangular elements being the ones that get better results in terms of optimization. While there are several studies to work with arbitrary-shaped elements, there is still much work to do in this field, and some interesting structuring elements, such as disks or ellipses, are difficult to decompose or to be approximated by polygons.

As a consequence, in this work, we show a new mathematical morphology approach, the morphological trajectory model (MTM), which takes into account the trajectory in which a morphological operation is applied. As it will be shown, our method is independent from the structuring element size and can be easily applied to industrial systems and high-resolution images. To complete our task, in Section 2 we shall define the so-called trajectory-based morphological operators. Then, in Section 3, the computation of the trajectory-based filters is shown and, afterwards, Section 4 considers some of the experiments completed to verify that our system behaves properly. Finally, some important remarks to our work, as well as some future research tasks, are summarized in Section 5.

## 2. The Morphological Trajectory Model

The classical morphological model has a nondeterministic nature, as it is defined over elements of a set without order restrictions in the access to these elements. In our new approach, the morphologic operations will be restricted to support an order. The order of the morphology operation is important because it will represent the structuring element trajectory. As a result, in this section an order relation between the elements in a set will be included, so that a sequence of operations could be established and, therefore, a deterministic component will be added to the morphological paradigm. Let us define some terms first.

### 2.1. Preliminary Definitions

Let *E* be the domain where the sets to be treated are defined. Let us assume that, in general, *E* ≡ *R* ^*n*^. Let *X*⊆*E* be a subset of *E*. Thus, in the case of two-dimensional objects *E* ≡ *R* ^2^ and for three-dimensional objects *E* ≡ *R* ^3^ and, consequently, *X* would be a two-dimensional or three-dimensional object, respectively. Notice that the domain is defined in a real space, so the method is suitable for any continuous domain. Images can be considered a particular case, where the domain is discretized.

Let In⁡(*X*) be a function that obtains the inner part of a set (i.e., an object); that is, its result is object *X* without its borders. This function is defined as the set of positions of the center of a solid *n*-ball of radius *ε* so that the ball is inside the object:

(1) $$\begin{array}{c} \mathrm{In}\left(X\right)=\left\{x\in \frac{E}{\exists \varepsilon }>0:B\left(x,\varepsilon \right)\subset A\right\}, \end{array}$$

where *B*(*x*, *ε*) is a solid *n*-ball of center *x* and radius *ε*.

On the other hand, let Fr(*X*) be a function relating a set to its border, so that all the points belonging to the object contour are obtained:

(2) $$\begin{array}{c} \text{Fr}\left(X\right)=X-\mathrm{In}\left(X\right). \end{array}$$

**Figure 1:**
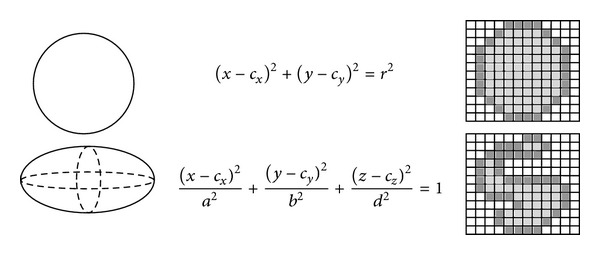
Examples of several possible representations of structuring elements. The leftmost figures show the analytical expressions of SEs; on the right, the corresponding classical SEs are shown.

As mentioned before, structuring elements are an essential tool to develop morphological operators. For methods that use classical mathematical morphology, the structuring element (SE) can be seen as a group of pixels. However, in our trajectory-based approach, the structuring element will be defined on the basis of the geometric definition of its frontier, so that any representation of the SE that allows the extraction of its frontier is valid for our method. An especially interesting case is the use of analytical expressions to define the SE, because this continuous representation gives, as a result, an adaptive precision and a more efficient computation than classical SE definitions, as it will be shown in the following sections.  Figure 1 illustrates this concept with two cases of analytical SEs (left) and classical SEs (right). For instance, the first row shows a circular SE, which can be analytically expressed as (*x*−*c*_*x*_)^2^ + (*y*−*c*_*y*_)^2^ = *r* ^2^, corresponding to a circumference centered on point (*c*_*x*_, *c*_*y*_) of radius *r*, instead of the classical neighborhood of pixels defined by the area of the circle, as shown on the right. This fact can be also extended to three-dimensional SEs, as shown on the second row in Figure 1.

This equation-based definition for the structuring element is used here for simplicity, but notice that our approach can be extended to any other frontier-based definition. Though the use of analytical or classical SEs in our model does not add any restriction for computing the trajectory-based morphological operators (as it will be shown, the method is based on a distance calculation), the analytical expression is preferable for efficiency and precision reasons.

### 2.2. Instant Basic Operations

A morphological operation will be divided into a sequence of unitary or* basic* operations. This sequence will guarantee the resulting order of the whole operation. Since every basic operation will correspond to a particular position of a structuring element along a trajectory that is performed during a period of time, we call them* instant basic operations*.

Let us define the instant basic operator ⊙_Γ(*k*)_, for any given instant *k*, as follows:

(3) $$\begin{array}{c} X{⊙}_{\Gamma (k)}B=p\in E:p={\mathrm{dist}}_{\vec{v}}\left(B,X•\Gamma \left(k\right)\right)\cdot \vec{v}\wedge B_{p}\cap X\neq Ø, \end{array}$$

where *X* is the target object, *B* the structuring element, *B*_*p*_ are copies of the structuring element centered at every point *p* when it touches the boundary of *X*, *E* ≡ *R* ^*n*^, Γ(*k*) represents an homogeneous transformation matrix in *R* ^*n*+1^ × *R* ^*n*+1^ obtained for a particular real value *k*, and ${\mathrm{dist}}_{\vec{v}}$ is the Euclidean distance between the structuring element and the transformation of the object *X*-obtained by postmultiplying every element of the set *X* by the homogeneous transformation matrix Γ(*k*)-computed in the direction addressed by vector $\vec{v}$. In other words, this operation obtains the structuring element center when it touches the boundary *X* following direction $\vec{v}$.

**Figure 2:**
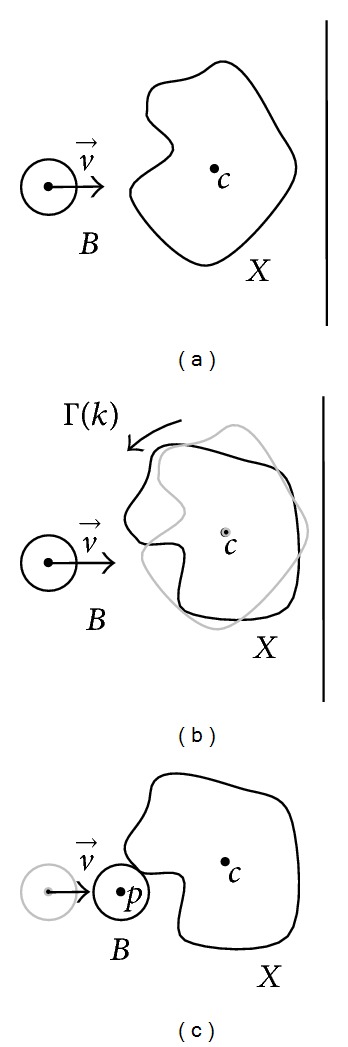
Geometric description of an instant basic operation. (a) Initial position. (b) Transformation of object *X*. (c) Distance computing.

A graphical example of this operator is shown in Figure 2. Thus, an object *X* is transformed applying a 2D rotation matrix over its center *c*. For this case, *k* could represent the number of degrees in that transformation matrix, so its values are in the [0,2*π*) range. Once the object is transformed (Figure 2(c)), the distance between *B* and *X* in the direction $\vec{v}$ is applied to the center of *B* in order to obtain the result of the instant basic operation (i.e., point *p*). For different and ordered real *k* values (using the < relation in *R*), we will obtain a new set of structuring element centers *B*_*p*_ that touch the boundary of *X*. These centers will also be ordered in the geometric space due to the use of different rotation matrixes.

The calculation of function ${\mathrm{dist}}_{\vec{v}}$ is the most time-consuming operation in (2). Here, the description of the structuring element plays a crucial role. We propose three methods for obtaining the distance ${\mathrm{dist}}_{\vec{v}}$ between the structuring element and the target object in the direction addressed by vector $\vec{v}$

- In the case of having an analytical description for both the SE and the target object, an analytical expression may be obtained for calculating the distance, as well. This way, the distance calculation is straightforward and its computation time will be low.
- When no object can be described using an analytical expression (neither the SE nor the target object), the distance is obtained using a discrete method: both objects must be discretized to obtain the points in their boundaries and the distance is obtained point-to-point. This is the worst case, and the computational cost depends on the discretization precision.
- Finally, a mixed method is proposed when only one object (the SE or the target object) can be described using an analytical expression. In this case, the distance is obtained between the points in the surface of the discretized object and the other object as a whole, using its analytical description. The computational cost is much lower than for the purely discrete method.

### 2.3. Trajectory-Based Morphological Operators

In this section, the instant basic operator ⊙_Γ(*k*)_ is applied in order to achieve a whole morphologic operation. We are particularly interested in defining the two fundamental operations in the morphologic paradigm: the dilation and the erosion. Due to the fact that frontiers of objects and structuring elements are only taken into account to compute the instant basic operations, the goal is to obtain only the boundary of dilation and erosion.

#### 2.3.1. Dilation

In general terms, this operation is classically defined as the place of the center positions of the structuring element *B* when it touches a set *X* [18]:

(4) $$\begin{array}{c} X⊕B=\left\{x\in E,B_{x}\cap X\neq Ø\right\}. \end{array}$$

In this expression, *B*_*x*_ is the translation of *B* so as to have its origin in point *x* ∈ *E*.

In our context, we are interested only in the dilation of the boundary, Fr(*X* ⊕ *B*), which is the place of the center positions that touch the boundary *X*:

(5) $$\begin{array}{c} \text{Fr}\left(X⊕B\right)=\left\{x\in E,B_{x}\cap X\neq Ø\wedge B_{x}\cap \mathrm{In}\left(X\right)\neq Ø\right\}. \end{array}$$

Derived from (3) and (5), we define the* instant basic dilation*, which specifies a center position touching the boundary of a set *X*, but from the outside:

(6) X⊕Γ(k)B=p∈E:p=dist⁡v→(B,X•Γ(k))·v→∧Bp∩X≠Ø∧Bp∩In⁡(X)≠Ø.

Using the instant basic dilation, we define the* trajectory-based dilation*, *X*⊕_Γ_ *B*, as the set of points resulting from the repeated and ordered application of the instant basic dilation, for the normalized *k* range [0,…, 1]. The trajectory, defined by Γ(*k*), must cover all the surface of the object in the normalized range. Note that only boundary points are computed and that the frontier of the trajectory-based dilation is expressed in (6):

(7) X⊕ΓB=⋃k∈[0,…,1](X⊕Γ(k)B)={x∈E,Bx∩X≠Ø∧Bx∩In⁡(X)=Ø}.

Trajectory-based dilation can orientate the structuring element in any position on the object boundary by means of homogeneous transformations, which are a combination of translations and rotations. This feature is not supported by classical dilations. In addition, partial dilations of objects are now also possible when a subrange of *k* is chosen.

#### 2.3.2. Erosion

Classically, this operation—which is commonly used for image filtering—is defined as the place of the center positions of the structuring element *B* when it is forced to be inside a set *X*:

(8) $$\begin{array}{c} X\Theta B=\left\{x\in E,B_{x}\subseteq \mathrm{In}\left(X\right)\right\}. \end{array}$$

In our context, we are interested only in the erosion boundary, which is the place of the center positions that touch the frontier of set *X* from the inside:

(9) $$\begin{array}{c} \text{Fr}\left(X\Theta B\right)=\left\{x\in E,B_{x}\subseteq \mathrm{In}\left(X\right)\wedge B_{x}\cap \text{Fr}\left(X\right)\neq Ø\right\}. \end{array}$$

Derived from (3) and (9), we define the* instant basic erosion*, which specifies a center position touching the boundary of a set *X*, but from the inside:

(10) XΘΓ(k)B=p∈E:p=dist⁡v→(B,X•Γ(k))·v→∧Bp⊆In(X)∧Bp∩Fr(X)≠Ø.

Consequently, we define the* trajectory-based erosion*, *X*Θ_Γ(*k*)_ *B*, as the set of points resulting from the repeated and ordered application of the instant basic erosion for the normalized *k* range [0,…, 1]. As in the case of dilation, the trajectory, defined by Γ(*k*), must cover all the surface of the object in the normalized range. The frontier of the trajectory-based erosion is

(11) XΘΓB=⋃k∈[0,…,1](XΘΓ(k)B)={x∈E,Bx⊆In⁡(X)∧Bx∩Fr(X)=Ø}.

**Figure 3:**
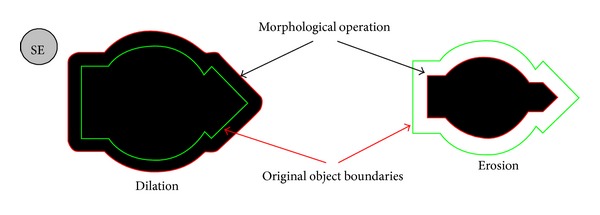
Classical morphological operations on 2D images. On the left, a morphological dilation. On the right, a morphological erosion. The structuring element (SE)—a circle of 20 pixels in radius—is shown at the top left corner.

Figure 3 shows an example of dilation and erosion applied to a 2D image. The black part corresponds to the classical operation result. The frontier is computed by means of the associated trajectory-based operator.

**Figure 4:**
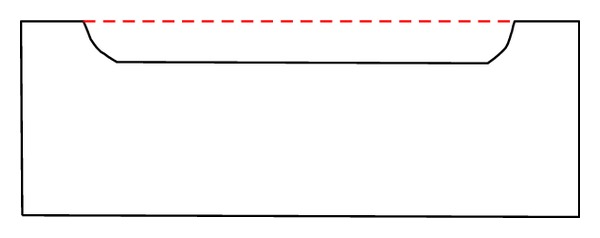
Partial morphological erosion as a subset of the complete erosion (over the subrange defined by the dotted line).

As with dilation, trajectory-based erosion can orientate the structuring element in any position on the object boundary, and define partial erosion of objects when a subrange of *k* is chosen (see Figure 4).

Table 1 summarizes the main differences between the methods described in this section. In classical mathematical morphology, the calculation is made on a complete image; that is, the morphological operation does not distinguish whether the pixels belong to a specific object or not; it simply applies a calculation operation of supremum or infimum in a neighborhood environment. In the morphological trajectory model (MTM), it is necessary to differentiate between the objects given in the space, since each object has a different geometric representation. Furthermore, this representation defines the frontier of the object and not its interior. Another important difference is the representation of the structuring element, whereas in traditional morphology it is treated as a subgroup of points (which is discretized for the case of working with images), the MTM considers the geometric function of the points that make up its frontier, without being necessary to carry out a discretization of the structuring element.

## 3. Morphological Trajectory Computation

In this section, the computation of the erosion for the MTM is presented. The algorithm becomes straightforward if the morphological erosion concept defined in [1] is applied. First of all, the boundary curve *C* of object *X* is represented as a set of ordered points *p* organized in collinear segments *s*. For every point, we compute the structuring element center position (*p*′) that touches each point in the direction of a certain vector ${\vec{v}}_{p}$, which must be perpendicular from inside object *X*. The center *p*′ will be valid only if the structuring element placed at *p*′ is inside the shape (i.e., it will not collide with the curve *C*). Note that for the dilation operation the procedure will be the same, but in this case the element will touch the boundary from the outside. A pseudo code algorithm for the erosion is presented in Algorithm 1.

**Figure 5:**
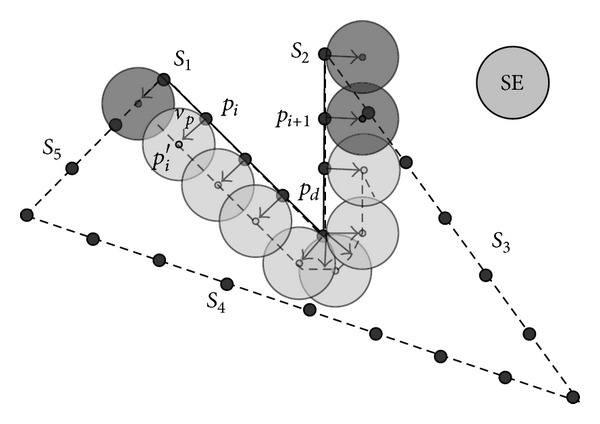
Analysis of segments *S*_1_ and *S*_2_ of a five-segment shape *C*. Dark-grey SE positions are discarded due to shape collision. Note that discontinuity at *p*_*d*_ is solved by a vector swept generation.

If a point *p* presents a discontinuity in the first derivative, we generate a set of new vectors in order to cover the gap (see Figure 5). From that new set we also compute new possible structuring element centers.

Let us analyze now the computational cost of the MTM algorithm in terms of the problem size. The operator used is *O* to determine an upper limit of the computation cost. As shown in Algorithm 1, the algorithm essentially consists of an external loop, which is used to have access to every point of the shape and two main function calls. Let's call *n* to the number of points that represent the shape *C* once it has been discretized. If we use a constant step factor *s* and the total length of *C* is *L*, then *n* will be *L*/*s* points.

The function* ObtainSECenter* computes the center of the structuring element when it touches a point *p* in the direction addressed by vector ${\vec{v}}_{p}$. So, this function depends on the SE geometry. For simple SEs, such as circles, rectangles, and triangles, the function can be evaluated in a constant time* ct*. Equation (12) shows this function for a circular SE of radius *R*:

(12) $$\begin{array}{c} \text{ObtainSECenter}\left(p,{\vec{v}}_{p}\right)=p+R\cdot {\vec{v}}_{p}. \end{array}$$

The next function, called CollideSE(*p*′, *C*), is true if the SE centered at point *p*′ is not completely inside shape *C* and returns false otherwise. In order to evaluate this condition, this function computes the intersection of the SE geometry and shape *C*. The cost of this function depends on the representation of *C*. For the experiments, we have organized the shape into a set of contiguous segments that represents the shape. Then, every segment is tested (at a constant time) and if a segment produces two or more intersections in the SE geometry then the function returns a true value. Note that, in this case, the discretization of shape *C* will not be the same as the one we used to determine the center positions in the shape. For shapes with a high degree of colinearity, the number of segments will be reduced slightly. Let us call *m* that number of segments.

The expressions in Table 2 show the quadratic equation used to determine the intersection between a circle centered at the origin and a 2D segment defined between points (*x*_1_, *y*_1_) and (*x*_2_, *y*_2_) for a normalized range *t*  [0,…, 1]. As a conclusion, a double solution for variable *t* in the range [0,…, 1] will cause a true return in the function* CollideSE*, otherwise the next segment will be analyzed.

Finally, the third function, called* AddTrajectory*, adds the new center *p*′ to the list of successful centers at a constant time, so it is not considered for evaluating the cost.

As a conclusion, let us analyze the whole algorithm in order to obtain an upper limit for the computational cost. The next expression evaluates this cost:

(13) $$\begin{array}{c} \underset{n,m\to \infty }{\lim}\left(n\cdot \left(ct+m\right)\right)=O\left(n\cdot m\right). \end{array}$$

We must remark that after completing our experiments, *m* ≪ *n* in most cases, since a usual value for *m* takes values of hundreds. The computation times for some examples of the MTM operations will be shown in the following section.

## 4. Experiments

In this section, we present some experiments in order to test the trajectory-based operations. The first one compares two versions of the classical dilation versus the trajectory-based one. In Algorithm 2, we show the classical version algorithms used in the tests.

The algorithm called MM1 corresponds to a classical morphological dilation, whereas the MM2 refers to that classical version operating only on the boundary of the object. Note that MM2 does not perform a valid dilation. It was only developed to test the* frontier* effect, that is, the advantage that MTM has since it only processes boundary pixels. The trajectory-based version was called MTM for the experiments. The images were evaluated on an Intel Pentium Dual Core processor @ 2.8 GHz and 2 GB in RAM. They were obtained on a Windows based platform.

Several tests and experiments were carried out in order to obtain the computing time under different input conditions. Both the size of the object and the size of the structuring element were varied, as well as the parameters that took part in the morphological operation.

**Figure 6:**
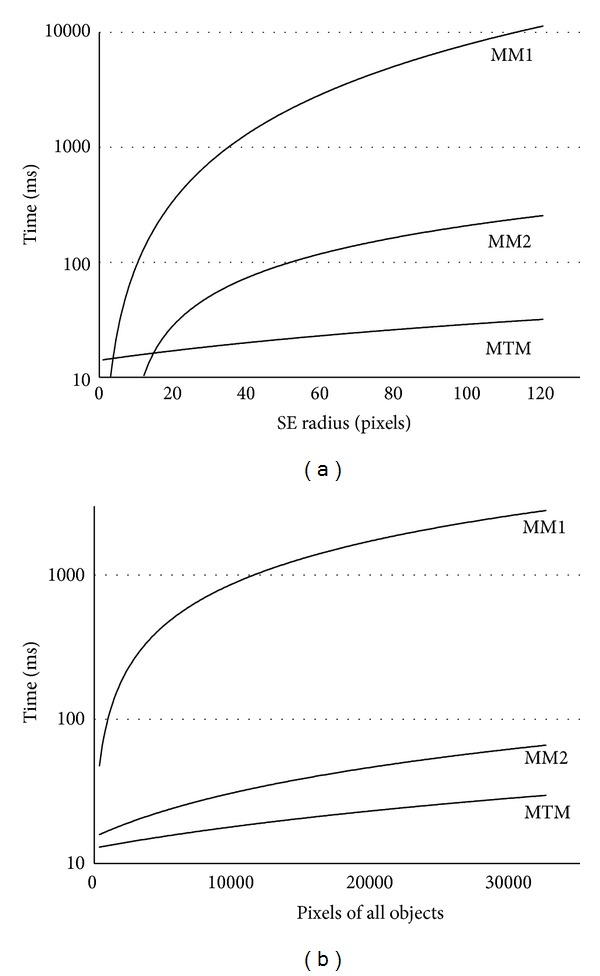
Morphological dilation tests. On the left, influence of the size of the structuring element. On the right, influence on the size of objects.

Figure 6 shows the behavior of the algorithms resulting from the variation of the size of the structuring element and the size of the object, respectively. As a consequence, from these experiments we can see that the computing time of the morphological trajectory model remains almost constant against the variations in the size of the structuring element; this is logical due to the fact that we use its geometric representation instead of its content, as opposed to MM1 and MM2. The difference between MM2 and MM1 arises from the fact that the former only expands the structuring element for the pixels in the object boundary and, therefore, its computing time decreases an order of magnitude with respect to MM1.

With regard to the variation in the size of the objects, in this experiment the MTM gives better results than MM2 and much better than MM1. Since MM2 works only on the object boundary, the difference with the MTM arises from the size chosen for the SE: if this size is small enough, MM2 will employ less time than the MTM as it has a simpler computational logic (see Algorithm 2). The radius of the structuring element chosen for this test was 40 pixels, which is equivalent to an area of 5025 points. In order to determine the effect of the increase of the size of the structuring element on a group of objects, the experiments described below were carried out.

**Figure 7:**
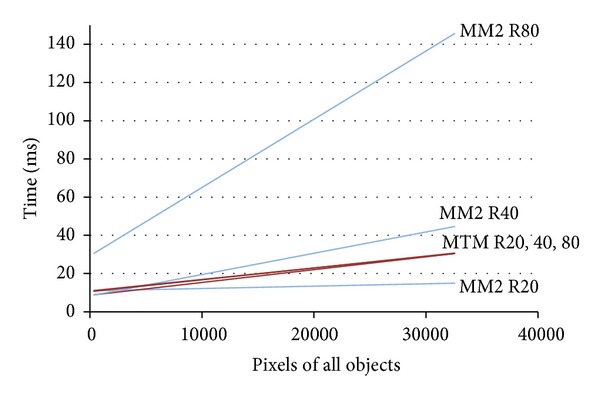
Morphologic dilation. Comparative study between MM2 and MTM models for SE sizes of 20, 40, and 80 pixels.

The experiment shown in Figure 7 compares the execution times of the MTM and MM2 for different radii of the SE. It may be seen that the increase in the structuring element size causes a crossing point between MM2 and MTM, for object sizes of about 5000 pixels.

Furthermore, Figure 7 shows that there is no significant increase in the computation time for the MTM when the size of the structuring element is increased, since this variation is minimal compared to the classical morphological methods. The small increase comes from the number of pixels of the object where the distance function is defined. Thus, if the size of the SE increases, this number is greater, and this causes the need to make new calculations on the new points.

**Figure 8:**
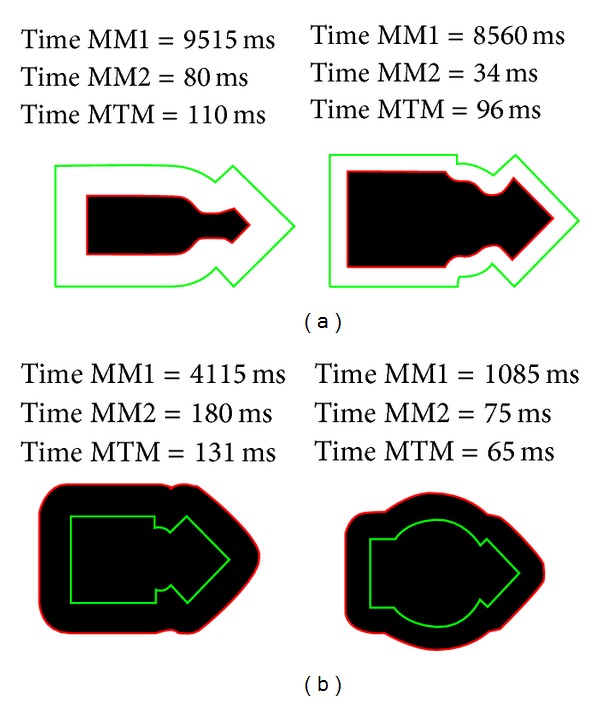
Morphological dilation and erosion. Results of different morphological operations used for the experiments. In the upper part, two erosions and in the lower part, two dilations. The boundary of the original object is represented in green and the result of MM1 operation in black, with the MTM result in red.

On the other hand, Figure 8 shows different results of the application of the morphological filters. Specifically, it is shown how the MTM obtains the frontier of the morphological operation made by MM1.

**Figure 9:**
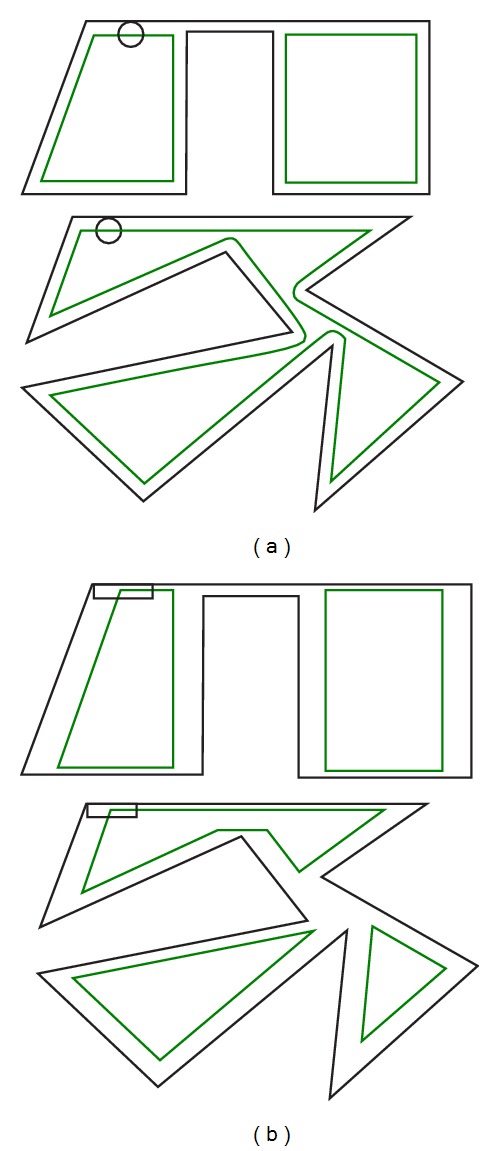
Erosion of figures using our morphological approach. (a) Using a circle as a structuring element. (b) Using a rectangle as a structuring element.

Finally, in Figure 9 we present some images related to other erosion experiments using several structuring element geometries, where the result is presented in green.

## 5. Conclusions and Discussion

In this paper, we have developed a topological system, resulting from applying the conventional morphological model, by means of trajectory-based morphological operations; to do this, we introduced a new feature, consisting of ordering the morphological primitives. As shown, the proposed operations are especially useful when large images need to be processed.

The morphological trajectory model offers an effective alternative to traditional methods for computing morphological primitives. This alternative is justified if the number of points of the objects and that of the structuring elements are high. The independency from the structuring element size could be interesting to apply morphological operations on high definition images or 3D image reconstruction. Due to the fact that the number of the group points is directly related to the dimension of the space in which the object and the structuring element are defined, the importance of the MTM is more relevant when the dimension of the representation space is increased (3D, 4D,…). In the two dimensional space, the application of the MTM may be justified for high-resolution images where large size operators are applied.

Other trajectory-based operations, such as openings, closings, and skeletons, are defined in [18]. We are interested in demonstrating their utility and efficiency by means of this trajectory optimization.

The new model has been presented for binary 2D images. However, the new paradigm is extensible for any number of dimensions of the Euclidean space. In [19–21], new versions of the morphological model for color images were presented. These models consider the color as a third coordinate and the computation is made in the 3D space. Future works will be focused on extending the model in order to support efficient filtering of color images or real 3D images.

## Figures and Tables

**Algorithm 1:**
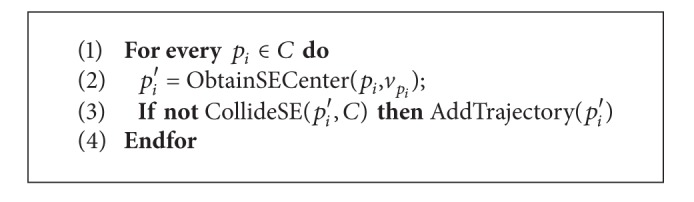
Basic pseudo-code algorithm for the morphological trajectory erosion.

**Algorithm 2:**
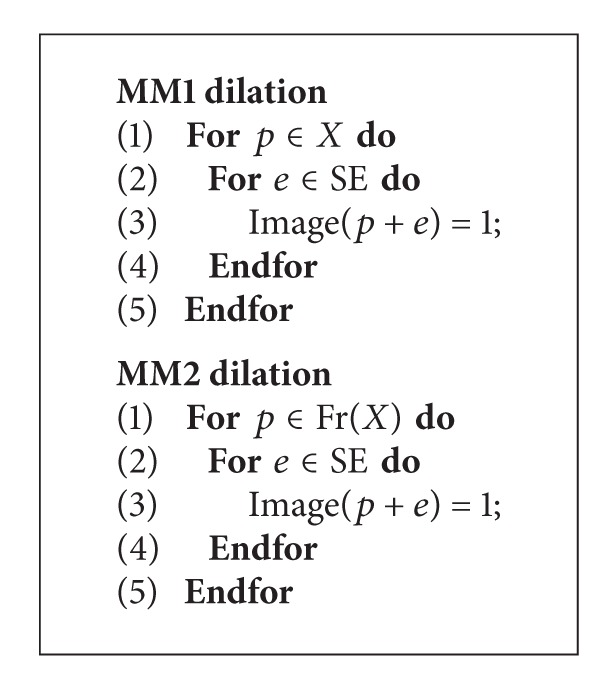
Pseudo-code used for the 2D experiments.

**Table 1:** Characteristics of the algorithms based on classical mathematical morphology versus the morphological trajectory model.

	Classical morphology	Morphological trajectory model
Application space	Finite group of pixels as a discretization of Euclidean space	2D Euclidean space (extensible to *n*-D Euclidean space)

Objects	Based on a complete image	Based on the boundary of each object (discrete/continuous)

SEs	Group of pixels	Geometric representation of the frontier

Method	On each pixel it operates in a neighborhood environment defined by the SE	The minimum distance of the SE center is calculated in a direction $\vec{v}$ on a trajectory defined by Γ(*k*)

Result	Erosion/dilation as group of pixels	Frontier points of the erosion/dilation operation

**Table 2:** Equations to calculate the intersection between a circle and a 2D segment.

Segment function	Circle function	Segment-Circle intersection equation on *t*
$\begin{array}{c} x=x_{1}+(x_{2}-x_{1})\cdot t\\ y=y_{1}+(y_{2}-y_{1})\cdot t\\ t\in [0,\ldots ,1] \end{array}$	*x* ^2^ + *y* ^2^ = *R* ^2^	(*x*_1_ + (*x*_2_ − *x*_1_) · *t*)^2^ + (*y*_1_ + (*y*_2_ − *y*_1_) · *t*)^2^ = *R* ^2^

## References

[1] Serra J (1982). *Image Analysis and Mathematical Morphology*.

[2] van Herk M (1992). A fast algorithm for local minimum and maximum filters on rectangular and octagonal kernels. *Pattern Recognition Letters*.

[3] Gil J, Werman M (1993). Computing 2-D min median and max filters. *IEEE Transactions on Pattern Analysis and Machine Intelligence*.

[4] Gil JY, Kimmel R (2002). Efficient dilation, erosion, opening, and closing algorithms. *IEEE Transactions on Pattern Analysis and Machine Intelligence*.

[5] Déforges O, Normand N, Babel M (2013). Fast recursive grayscale morphology operators: from the algorithm to the pipeline architecture. *Journal of Real-Time Image Processing*.

[6] Clienti C, Bilodeau M, Beucher S, Blanc-Talon J, Bourennane S, Philips W, Popescu D, Scheunders P (2008). An efficient hardware architecture without line memories for morphological image processing. *Proceedings of the 10th International Conference on Advanced Concepts for Intelligent Vision Systems (ACIVS '08)*.

[7] Sivakumar K, Patel MJ, Kehtarnavaz N, Balagurunathan Y, Dougherty ER (2000). A constant-time algorithm for erosions/dilations with applications to morphological texture feature computation. *Real-Time Imaging*.

[8] Soille P, Talbot H, Jain A, Venkatesh S, Lovell B Image Structure Orientation Using Mathematical Morphology.

[9] Soille P, Talbot H (2001). Directional morphological filtering. *IEEE Transactions on Pattern Analysis and Machine Intelligence*.

[10] Urbach ER, Wilkinson MHF (2008). Efficient 2-D grayscale morphological transformations with arbitrary flat structuring elements. *IEEE Transactions on Image Processing*.

[11] Zhang Y, Wu L, Tan Y, Shi Y, Chai Y, Wang G (2011). Recursive structure element decomposition using migration fitness scaling genetic algorithm. *Proceedings of the 2nd International Conference on Advances in Swarm Intelligence (ICSI '11)*.

[12] Anelli G, Broggi A, Destri G (1998). Decomposition of arbitrarily shaped binary morphological structuring elements using genetic algorithms. *IEEE Transactions on Pattern Analysis and Machine Intelligence*.

[13] Park H, Yoo J (2001). Structuring element decomposition for efficient implementation of morphological filters. *IEE Proceedings: Vision, Image and Signal Processing*.

[14] Shih FY, Wu Y-T (2005). Decomposition of binary morphological structuring elements based on genetic algorithms. *Computer Vision and Image Understanding*.

[15] Hedberg H, Dokladal P, Öwall V (2009). Binary morphology with spatially variant structuring elements: algorithm and architecture. *IEEE Transactions on Image Processing*.

[16] Dokládal P, Dokládalová E (2011). Computationally efficient, one-pass algorithm for morphological filters. *Journal of Visual Communication and Image Representation*.

[17] van Droogenbroeck M, Buckley MJ (2005). Morphological erosions and openings: fast algorithms based on anchors. *Journal of Mathematical Imaging and Vision*.

[18] Cooksey E, Withers WD Rapid image binarization with morphological operators.

[19] Goutsias J, Heijmans HJAM, Sivakumar K (1995). Morphological operators for image sequences. *Computer Vision and Image Understanding*.

[20] Hanbury A Mathematical morphology in the HLS colour space.

[21] Angulo J, Serra J Morphological color size distribution for image classification and retrieval.

